# Role of modified hydration for preventing contrast-associated acute kidney injury in patients with ST-segment elevation myocardial infarction after primary percutaneous coronary intervention

**DOI:** 10.1007/s11739-022-03109-3

**Published:** 2022-12-20

**Authors:** Lei Liu, Li Zhou, Weiping Li, Hui Chen, Hongwei Li

**Affiliations:** 1 Department of Cardiology, Cardiovascular Center, Beijing Friendship Hospital, Capital Medical University, 95 Yongan Road, Beijing, 100050 China; 2 Beijing Key Laboratory of Metabolic Disorder Related Cardiovascular Disease, Beijing, China; 3 Department of Geriatrics, Cardiovascular Center, Beijing Friendship Hospital, Capital Medical University, Beijing, China

**Keywords:** Hydration, Contrast-associated acute kidney injury, Percutaneous coronary intervention, Major adverse cardiovascular events

## Abstract

To assess the efficacy of modified hydration on contrast-associated acute kidney injury (CA-AKI) in ST-segment elevation myocardial infarction (STEMI) after primary percutaneous coronary intervention (pPCI). A total of 438 patients were randomly assigned to 2 groups. The traditional hydration group (group I) was given at a rate of 1 ml/kg/h for 24 h, and the modified hydration group (group II) was given at a rate of 3 ml/kg/h in the first 4 h, and then reduced to 1 ml/kg/h for 12 h. 0.3 mg/kg of furosemide was given 1-h after hydration. The primary endpoint was the incidence of CA-AKI, and the secondary endpoint was the incidence of major adverse cardiovascular events (MACEs) during a median of 22.4 months (IQR 9.6, 32.6 months) follow-up. The incidence of CA-AKI was 8.7%. Among these, Group I was 9.1% and group II was 8.2%, respectively. There was no significant difference in CA-AKI and creatinine levels between the two hydration groups. Multivariable logistics regression analysis revealed that creatinine, white blood cells, and N-terminal pro-B-type natriuretic peptide were associated with CA-AKI. Moreover, CA-AKI was an independent predictor for all-cause death and cardiac death during the follow-up period. The modified hydration may reduce the incidence of CA-AKI, although this difference was not statistically significant. The relationship between CA-AKI and mortality strengthened as creatinine times above baseline increased. Mitigating the occurrence of CA-AKI may reduce all-cause death and cardiac death.

**Supplementary Information:**

The online version contains supplementary material available at 10.1007/s11739-022-03109-3.

## Introduction

The widespread adoption of primary percutaneous coronary intervention (pPCI) has significantly improved the cardiovascular outcomes of ST-segment elevation myocardial infarction (STEMI), however, the incidence of contrast-associated acute kidney injury (CA-AKI), which has replaced the definition of contrast-induced acute kidney injury (CI-AKI) in current guidelines [1, 2], may increase due to the emergency of STEMI, attendant hemodynamic compromise and the use of higher contrast volumes [3, 4]. A previous study has shown that the incidence of CA-AKI in patients with STEMI who underwent pPCI occurred in up to 30% [5]. CA-AKI can be preventable in those patients if the underlying risk factors are well identified and managed properly during the acute event [6]. Reducing the amount of contrast media as much as possible and using optimal hydration before and immediately after percutaneous coronary intervention (PCI) are recommended for reducing the incidence [7]. Notably, inadequate hydration markedly increases the incidence of CA-AKI, while excessive hydration may increase the risk of heart failure [8]. Therefore, the identification of optimal hydration dose for patients which can appropriately and effectively prevent CA-AKI is important. But there is no guidance or consensus on how to hydrate in patients undergoing pPCI.

The pharmacokinetics of the contrast agent showed that 80% of the contrast agent was excreted by the kidney in the first 4 h and the remaining 20% was excreted slowly within the next 24 h [8]. The hemodynamic response to intra-arterial injection of contrast agent is biphasic: a brief initial increase in renal blood flow followed by a prolonged decline of 10% to 25% below baseline [9]. Therefore, based on the pharmacokinetics of the contrast agent, we studied the effect of modified hydration in a prospective randomized controlled trial involving patients with STEMI undergoing pPCI.

## Methods

### Study population

A total of 508 STEMI patients undergoing pPCI at the Cardiovascular Center of Beijing Friendship Hospital from November 2018 to October 2021 were enrolled in this study. 70 patients were excluded according to the exclusion criteria including acute respiratory failure; continuous renal replacement therapy or prior kidney transplantation; uncontrolled congestive heart failure; exposure to contrast agent within 7 days before pPCI or 3 days after pPCI; malignant tumor; patients who did not complete hydration due to various reasons (Supplementary Fig. 1). Finally, consecutive 438 patients were included in the final analysis. All patients were followed up to December 2021 with a median follow-up of 22.4 months (IQR 9.6, 32.6 months).

The local institutional review board at our hospital approved the study protocol, and this study was in accordance with the Declaration of Helsinki.

### Study protocol

Eligible patients were randomly assigned to group I (traditional hydration group) and group II (modified hydration group). The traditional hydration group received a continuous intravenous infusion of isotonic saline at a rate of 1 ml/kg/h for 24 h at the beginning of pPCI [10]; the modified hydration group received a continuous intravenous infusion of isotonic saline at a rate of 3 ml/kg/h at the beginning of pPCI for 4 h. After 1 h of hydration, 0.3 mg/kg of furosemide was given for diuretic effect (except patients with systolic blood pressure < 90 mmHg). After 4 h, the rate was reduced to 1 ml/kg/h for 12 h. Equal intravenous hydration volume was given in the two groups. There was no further hydration treatment before and after the study protocol. Computer-generated random numbers determined randomization. All patients were treated with the same nonionic, equal osmolarity iodinated contrast medium during the procedure.

### Other data collection and definitions

Venous blood samples were taken from all patients pre-pPCI and then 24, 48 and 72 h after pPCI, samples were used for measurement of serum creatinine and estimated glomerular filtration rate (eGFR) based on Modification of Diet in Renal Disease (MDRD) equation in (ml/min/1.73m^2^). Patients’ demographic information, medical and medication history, and laboratory measurements were collected and confirmed through electronic medical records. The total ischemic time (the time from symptom initiation to reperfusion) was also collected [11]. The left ventricular ejection fraction (LVEF) was determined using 2-dimensional echocardiography during the index hospitalization.

STEMI was diagnosed based on the diagnostic criteria recommended by the European Society of Cardiology (ESC): (1) chest pain lasting more than 30 min.; (2) the dynamic electrocardiogram change: ST-segment elevation was measured at the J-point at least in two contiguous leads with ST-segment elevation of 2.5 mm in men < 40 years, 2 mm in men 40 years, or 1.5 mm in women in leads V2–V3 and/or 1 mm in the other leads in the absence of left ventricular hypertrophy or left bundle branch block; (3) levels of serum markers of myocardial injury were changed; (4) the coronary angiography showed that patients could receive PCI for anatomy in infarct-related artery[12]. The Killip score class on admission was determined based on chest radiograph findings, lung sounds on auscultation, and blood pressure. In brief, patients were classified into four categories: Killip class I, STEMI without heart failure; Killip class II, STEMI with mild to moderate heart failure (as the presence of rales and/or jugular venous distension); Killip class III, STEMI with pulmonary edema; and Killip class IV, STEMI with cardiogenic shock [13, 14].

### Coronary angiography and PCI

Patients with STEMI received coronary angiography immediately after admission to the catheter laboratory, with a digital subtraction machine. The coronary angiography was performed by cardiologists in Beijing Friendship Hospital, and all STEMI patients received PCI surgery as soon as possible after admission to open the infarct-related coronary artery. Meanwhile, two interventional cardiologists judged the stenosis of the coronary artery by a double-blind method.

### Study endpoints

The primary endpoint of the study was the incidence of CA-AKI, which identifies events based on Improving Global Outcomes (KDIGO) serum creatinine criteria [increase in serum creatinine by ≥ 26.5 µmol/l (0.3 mg/dl) or increase in serum creatinine by ≥ 1.5 times baseline value]. AKI was said to be present if these changes occurred within 72 h from the initial contrast dose [2, 15]. Stage 1 is characterized by an increase in serum creatinine of > 26.53 μmol/l (0.3 mg/dl) or 1.5 to 1.9 times increase above baseline; stage 2 by 2.0 to 2.9 times increase above baseline; and stage 3 by > 3 times increase above baseline, or an increase of > 353.68 μmol/l (4.0 mg/dl), or initiation of renal replacement therapy.

The secondary endpoint was the occurrence of major adverse cardiac events (MACEs) including all-cause death, cardiac death, non-fatal myocardial infarction (MI), coronary revascularization, and cardiac rehospitalization. All-cause death was defined as the incidence of cardiac death or non-cardiac death. Cardiac death was defined as fatal MI and heart failure, sudden death, and other cardiac death. Non-fatal MI was defined as chest pain with new ST-segment changes and elevation of myocardial necrosis markers to at least twice the upper limit of the normal range. Coronary revascularization was defined as the revascularization of the target vessel or non-target vessels. Cardiac rehospitalization referred to rehospitalization for angina pectoris or heart failure.

### Statistical analysis

Depending on the distribution of the data, continuous variables were expressed as mean value ± SD or median and interquartile range (IQR). Frequencies and percentages were used to describe categorical data. Differences between continuous and categorical variables were assessed using Student’s T-test, analysis of variance, Chi-square test, and repeated measures ANOVA as appropriate. Multivariable logistics regression analysis was conducted to analyze the independent risk factors for CA-AKI. Multivariable Cox regression analysis was conducted to analyze the independent risk factors for MACEs in STEMI patients after pPCI. The cumulative incidence of MACEs was estimated by Kaplan–Meier survival curves, and the groups were compared using the log-rank test. Hydration difference on secondary endpoint was assessed with analyses of P for interaction. All analyses were two-tailed and *P* value < 0.05 was considered statistically significant. Data were analyzed using SPSS statistical package version 26.0 (SPSS Inc., Chicago, IL, USA).

## Results

### Baseline characteristics in traditional and modified hydration group

As shown in Table 1, 438 eligible patients (mean age 60.8 years, men 76.9%) were included in our study, more than half of the patients (60.5%, *N* = 265) were identified as having hypertension, 28.3% (*N* = 124) had diabetes mellitus (DM), 5.5% (*N* = 24) had CHD, 2.3% (*N* = 10) had a history of MI. Compared with group I, group II showed a significantly higher percentage of current/ex-smokers, higher LVEF, lower percent of triple-vessel lesions, lower SYNTAX score, and lower α_1_-microglobulin and microalbumin.

**Table 1:** Clinical characteristics in different hydration groups

	Total	Group I	Group II	*P* value
	*N* = 438	*N* = 219	*N* = 219	
Male (%)	337 (76.9)	162 (74.0)	175 (79.9)	0.140
Age(years)	60.8 ± 12.6	61.0 ± 12.8	60.6 ± 12.4	0.736
BMI (kg/m^2^)	25.7 ± 3.5	25.8 ± 3.7	25.6 ± 3.3	0.446
SBP (mmHg)	127.4 ± 22.9	126.7 ± 24.6	128.2 ± 20.9	0.478
DBP (mmHg)	77.5 ± 15.7	76.8 ± 17.2	78.1 ± 13.9	0.375
Heart rate(bpm)	75 (64, 86)	75 (64, 86)	75 (64, 86)	0.752
Anterior MI (%)	203 (46.3)	104 (47.5)	99 (45.2)	0.632
Killip II-IV (%)	43 (9.8)	22 (10.0)	21 (9.6)	0.872
Cardiogenic shock (%)	26 (5.9)	17 (7.8)	9 (4.1)	0.106
Medical history				
Hypertension (%)	265 (60.5)	130 (59.4)	135 (61.6)	0.625
DM (%)	124 (28.3)	70 (32.0)	54 (24.7)	0.090
CHD (%)	24 (5.5)	11 (5.0)	13 (5.9)	0.675
OMI (%)	10 (2.3)	3 (1.4)	7 (3.2)	0.201
Dyslipidemia (%)	199 (45.4)	100 (45.7)	99 (45.2)	0.924
Current/ex-smoker (%)	288 (65.8)	134 (61.2)	154 (70.3)	0.044
Pharmacotherapy before admission				
Antiplatelet agent, %	50 (12.6)	30 (13.7)	25 (11.4)	0.603
ACEI/ARB, %	218 (49.8)	103 (47.0)	115 (52.5)	0.251
β-blocker, %	35 (8.0)	14 (6.4)	21 (9.6)	0.217
Statins, %	199 (45.4)	100 (45.7)	99 (45.2)	0.924
Metformin, %	33 (7.5)	18 (8.2)	15 (6.8)	0.587
Laboratory values				
WBC(× 10^9^/L)	9.3 (7.5,11.8)	7.3 (7.5,12.3)	9.3 (7.6,11.3)	0.617
Hemoglobin(g/l)	139.5 ± 19.1	137.3 ± 21.5	140.5 ± 17.9	0.162
HsCRP(mg/l)	5.6 (2.3,17.4)	6.6 (2.6,18.6)	4.9 (2.2,14.8)	0.199
HbA_1_C (%)	6.0 (5.5,7.0)	6.1 (5.5,7.4)	5.9 (5.5,6.9)	0.252
TC (mmol/l)	4.6 (3.9,5.3)	4.6 (3.9,5.2)	4.6 (3.9,5.4)	0.560
TG (mmol/l)	1.5 (1.1,2.2)	1.5 (1.1,2.2)	1.6 (1.2,2.2)	0.212
LDL-C(mmol/l)	2.7 (2.2,3.2)	2.7 (2.2,3.2)	2.8 (2.2,3.3)	0.440
HDL-C (mmol/l)	1.0 (0.9,1.1)	1.0 (0.9,1.1)	1.0 (0.9,1.2)	0.732
pCKMB(ng/ml)	99.2 (40.3,209.0)	103.0 (41.3,207.0)	94.4 (36.7,213.0)	0.490
pTNI(ng/ml)	46.4 (15.9,50.0)	49.9 (15.5,50.0)	42.4 (16.3,50.0)	0.648
pNT-proBNP(pg/ml)	1380.0 (648.0,2737.0)	1445.5 (785.8,2526.3)	1259.0 (522.0,3016.5)	0.392
Angiography values				
LM (%)	469 (11.0)	14 (6.4)	13 (5.9)	0.843
Triple-vessel (%)	2768 (64.6)	136 (62.1)	113 (51.6)	0.026
PCI (%)	418 (95.4)	211 (96.3)	207 (94.5)	0.360
Volume (ml)	110 (110,130)	110 (110,130)	110 (110,130)	0.591
LVEF	0.57 (0.51,0.63)	0.57 (0.49,0.62)	0.58 (0.52,0.63)	0.041
Total ischemic time (hour)	4.5 (3.0,6.0)	5.0 (3.0,6.0)	4.0 (3.0,6.0)	0.584
TIMI score	4.0 ± 2.0	4.0 ± 2.1	4.0 ± 1.9	0.812
GRACE score	145.9 ± 25.9	146.5 ± 31.3	145.3 ± 27.7	0.669
SYNTAX score	16.1 ± 6.3	16.9 ± 5.7	15.3 ± 6.7	0.009
α_1_-microglobulin (mg/dl)	1.0 (0.6,1.9)	1.1 (0.6,2.4)	0.9 (0.6,1.7)	0.028
Microalbumin (mg/dl)	1.4 (1.1,4.0)	1.7 (1.1,4.5)	1.2 (1.1,3.2)	0.008
Intake of fluids in 24 h (ml)	2301.5 ± 588.5	2286.7 ± 580.9	2316.5 ± 598.1	0.699
Urine volume in 24 h (ml)	1852.3 ± 717.4	1804.9 ± 653.2	1900.2 ± 776.7	0.310

Total ischemic time represent the time from symptom initiation to reperfusion. Group I represent traditional hydration; group II represent modified hydration

*BMI* body mass index, *SBP* systolic blood pressure, *DBP* diastolic blood pressure, *MI* myocardial infraction, *DM* diabetes mellitus, *CHD* coronary heart disease, *OMI* old myocardial infarction, *WBC* white blood cells, *hsCRP* hypersensitivity C-reactive protein, *HbA1C* glycosylated hemoglobin, *TC* total cholesterol, *LDL-C* low density lipoprotein cholesterol, *HDL-C* high density lipoprotein cholesterol, *ACEI* angiotensin-converting enzyme inhibitor, *ARB* angiotensin II receptor blocker, *NT-proBNP* N-terminal pro-B-type natriuretic peptide, *CKMB* creatine Kinase Isoenzyme-MB, *TNI* troponin I, *LM* left main trunk, *PCI* percutaneous coronary intervention, *LVEF* left ventricular ejection fraction

### Serum creatinine and eGFR in traditional and modified hydration group

As shown in Table 2, CA-AKI occurred in 38 patients, and the incidence was 8.7%, including 20 patients (9.1%) in group I and 18 patients (8.2%) in group II (*P* = 0.734). Of these, the 32 (7.3%) had stage 1, 4 (0.9%) had stage 2 and 2 (0.5%) had stage 3.

**Table 2:** Changes in creatinine and eGFR

	Total	Group I	Group II	*P* value
	*N* = 438	*N* = 219	*N* = 219	
Creatinine at admission (umol/l)	73.9 ± 27.0	73.7 ± 27.5	74.1 ± 26.5	0.867
Creatinine at 24-h after pPCI (umol/l)	76.2 ± 26.3	74.7 ± 26.2	77.7 ± 26.3	0.229
Creatinine at 48-h after pPCI (umol/l)	78.3 ± 25.4	77.4 ± 23.4	79.3 ± 27.3	0.417
Creatinine at 72-h after pPCI (umol/l)	80.4 ± 29.2	79.2 ± 28.1	81.6 ± 30.3	0.380
eGFR at admission (ml/min/1.73m^2^)	102.3 ± 39.9	102.4 ± 42.0	102.2 ± 37.7	0.969
eGFR at 24-h after pPCI (ml/min/1.73m^2^)	98.6 ± 38.0	100.3 ± 40.1	96.9 ± 35.8	0.353
eGFR at 48-h after pPCI (ml/min/1.73m^2^)	95.3 ± 35.6	96.0 ± 37.7	94.6 ± 33.5	0.690
eGFR at 72-h after pPCI (ml/min/1.73m^2^)	93.4 ± 35.2	94.4 ± 37.7	92.4 ± 32.6	0.547
CA-AKI (%)	38 (8.7)	20 (9.1)	18 (8.2)	0.734

Group I represent traditional hydration; group II represent modified hydration

*pPCI* primary percutaneous coronary intervention, *eGFR* estimated glomerular filtration rate, *CA-AKI* contrast-associated acute kidney injury

The baseline serum creatinine levels were 73.7 ± 27.5umol/l in group I and 74.1 ± 26.5umol/l in group II. The creatinine levels continuously increased at 24-h, 48-h and 72-h after pPCI both in the two groups. There were significant differences in creatinine at different time points in each hydration group (*P* < 0.001) (Supplementary Fig. 2A). However, hydration did not affect the serum creatinine levels between the two groups (all *P* > 0.05) (Table 2).

The baseline eGFR levels were102.4 ± 42.0 ml/min/1.73m^2^ in group I and 102.2 ± 37.7 ml/min/1.73m^2^ in group II (*P* = 0.969). After pPCI, eGFR levels continuously decreased at 24-h, 48-h and 72-h. There were significant differences in eGFR at different time points in each hydration group (*P* < 0.001) (Supplementary Fig. 2B). However, there was also no significant difference in eGFR between the two groups (all *P* > 0.05) (Table 2).

Based on KDIGO criteria, we further classified CA-AKI for analysis. Briefly, grade1 was characterized by an increase in serum creatinine of < 1.25 times increase above baseline; grade 2 was 1.25–1.4 times increase above baseline; grand 3 was 1.5–1.9 times increase above baseline; grade 4 was > 2.0 times increase above baseline. As shown in Supplementary Fig. 3, there were no statistical differences in the different grades between the two hydration groups.

### Baseline characteristics in CA-AKI and no CA-AKI group, and multivariable logistic regression analysis

Compared with the no CA-AKI group, the CA-AKI group showed a significantly lower percentage of male and current/ex-smokers, higher white blood cells (WBC), hypersensitivity C-reactive protein, and glycosylated hemoglobin, higher peak value of N-terminal pro-B-type natriuretic peptide(pNT-proBNP), peak value of troponin I, and urine α_1_-microglobulin (Supplementary Table 1).

Multivariable logistic regression analysis was conducted to identify risk factors related to the development of CA-AKI. The results revealed that creatinine (OR = 0.975, 95% CI: 0.952 to 0.999, *P* = 0.042), WBC (OR = 1.166, 95% CI: 1.034 to 1.315, *P* = 0.012) and NT-proBNP (OR = 1.013, 95% CI 1.004 to 1.022, *P* = 0.006) were associated with the incidence of CA-AKI (Table 3).

**Table 3:** Multivariable logistic regression analysis of CA-AKI

	Univariable		Multivariable	
	HR (95%CI)	P value	HR (95%CI)	*P* value
Age (years)	1.018 (0.991,1.046)	0.191		
Male (%)	0.328 (0.166,0.650)	0.001		
WBC(× 10^9^/L)	1.204 (1.089,1.332)	< 0.001	1.166 (1.034,1.315)	0.012
Creatinine (umol/l)	0.980 (0.961,0.999)	0.040	0.975 (0.952,0.999)	0.042
HbA_1_C (%)	1.244 (1.015,1.473)	0.011		
Modified hydration (%)	0.891 (0.458,1.735)	0.734		
NT-proBNP (per 100 pg/ml)	1.688 (1.054,2.704)	0.029	1.013 (1.004,1.022)	0.006
ACEI/ARB (%)	1.010 (0.519,1.965)	0.977		

*SBP* systolic blood pressure, *NT-proBNP*, N-terminal pro-B-type natriuretic peptide, *CA-AKI* contrast-associated acute kidney injury, *ACEI* angiotensin-converting enzyme inhibitor, *ARB* angiotensin II receptor blocker, *WBC* white blood cells, *HbA1C* glycosylated hemoglobin

### Kaplan–Meier analysis of secondary endpoint

**Fig. 1:**
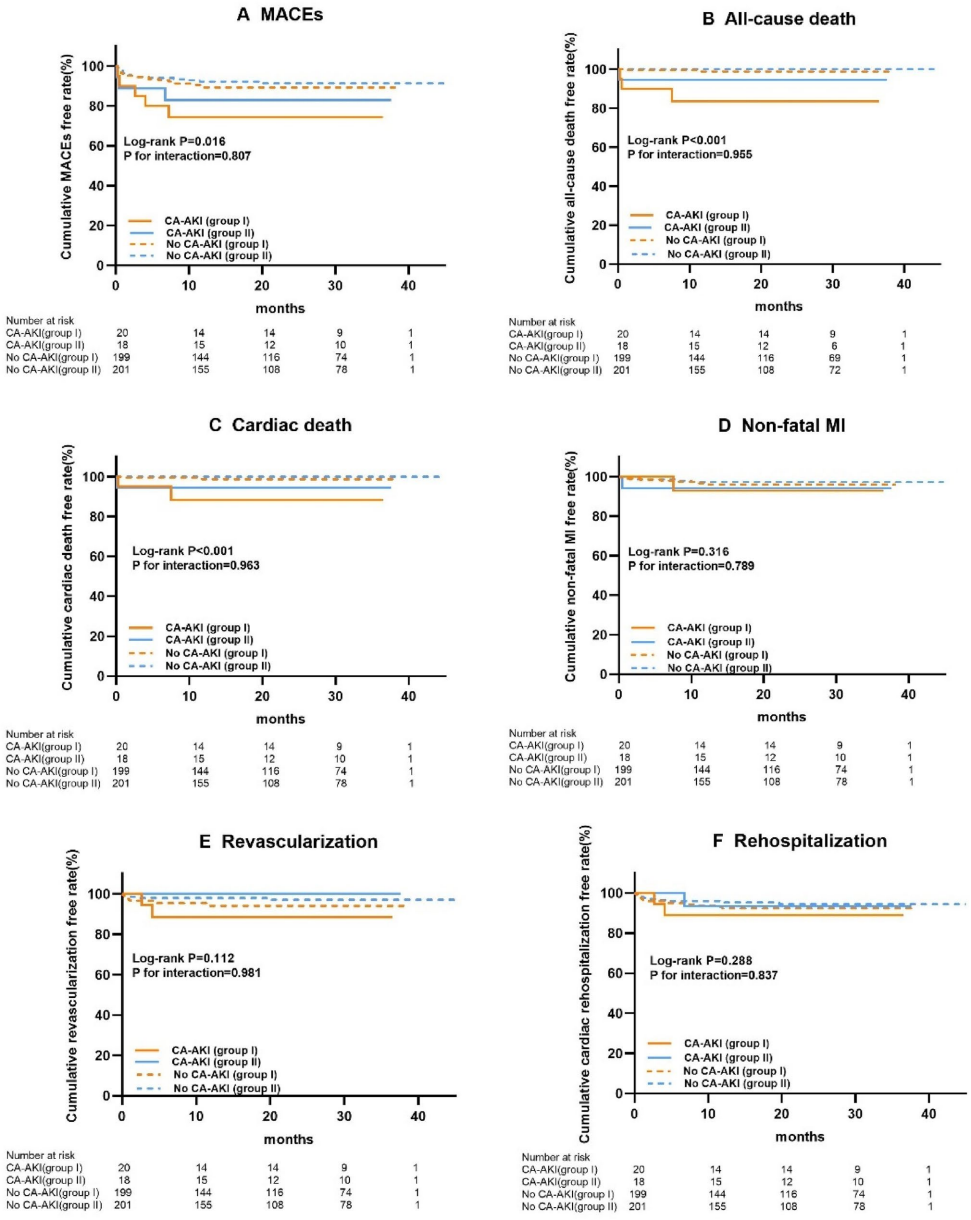
Kaplan–Meier curve for secondary endpoint. Group I represent traditional hydration; group II represent modified hydration. *MACEs* major adverse cardiac events, *MI* myocardial infraction, *CA-AKI* contrast-associated acute kidney injury

During a median follow-up period of 22.4 months, the incidence of MACEs was 21.1% in the CA-AKI group and 9.3% in the no CA-AKI group. The incidences of all-cause death and cardiac death were 10.5% and 7.9% in the CA-AKI group, 0.5% and 0.5% respectively in the no CA-AKI group (all *P* < 0.05). We also compared the secondary endpoint in different hydration groups, there was no statistically significant (Supplementary Table 2). The Kaplan–Meier curves show that the CA-AKI group had a significantly higher cumulative rate of MACEs, all-cause death and cardiac death than the no CA-AKI group (*P* = 0.006 vs. *P* < 0.001 vs. *P* < 0.001 respectively), and all P for interactions > 0.05 (Fig. 1).

### Independent association between MACEs, all-cause death and cardiac death

**Fig. 2:**
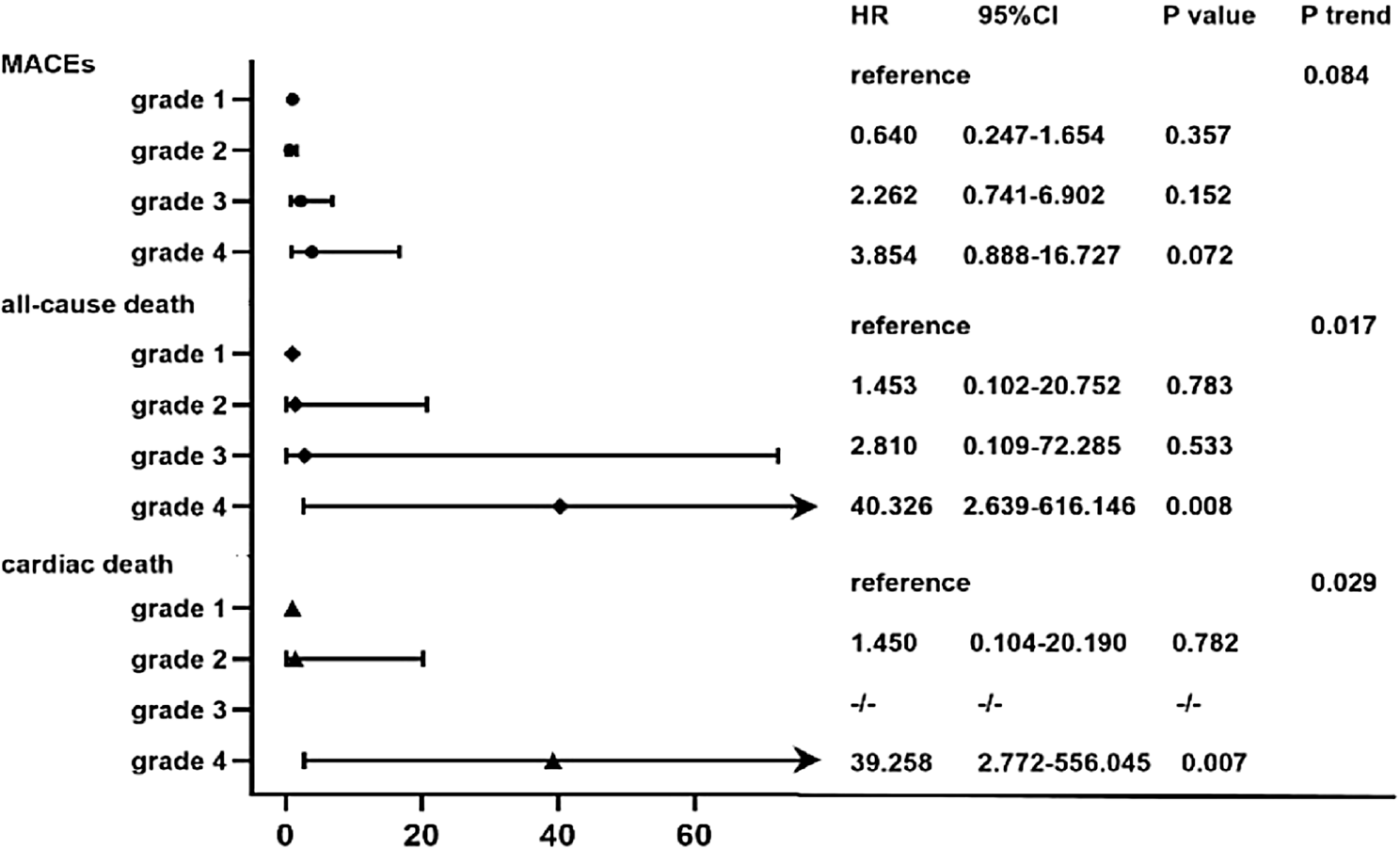
Creatinine grades and risk for development of secondary endpoints. *MACEs* major adverse cardiac events, *CA-AKI* contrast-associated acute kidney injury; N-terminal pro-B-type natriuretic peptide. Grade1 was characterized by an increase in serum creatinine of < 1.25 times increase above baseline; grade 2 was 1.25 to 1.4 times increase above baseline; grand 3 was 1.5–1.9 times increase above baseline; grade 4 was > 2.0 times increase above baseline. Model adjusted for sex, age, NT-proBNP, modified hydation and CA-AKI

In the multivariable Cox regression analysis, we included variables that were identified to be significantly associated with MACEs, all-cause death and cardiac death in the univariable model. The multivariable Cox regression analysis revealed that CA-AKI was significantly and independently associated with all-cause death and cardiac death. The relationship between CA-AKI and mortality strengthened as creatinine times above baseline increased (Fig. 2). However, after multivariable adjustment, CA-AKI was not associated with MACEs in our study cohort (Table 4 and Fig. 2).

**Table 4:** Multivariable Cox regression analysis of MACEs, all-cause death and cardiac death

	MACEs				All-cause death				Cardiac death			
	Univariable		Multivariable		Univariable		Multivariable		Univariable		Multivariable	
	HR (95%CI)	*P* value	HR (95%CI)	*P* value	HR (95%CI)	*P* value	HR (95%CI)	*P* value	HR (95%CI)	*P* value	HR (95%CI)	*P* value
Age (years)	1.026 (1.001,1.052)	0.039			1.152(1.056,1.258)	0.002			1.141(1.040,1.251)	0.005		
Male (%)	1.013 (0.500,2.049)	0.972			0.596(0.109,3.255)	0.550			1.197(0.134,10.710)	0.872		
Modified hydration (%)	0.750 (0.413,1.362)	0.345			0.197(0.023,1.688)	0.138			0.246(0.028,2.202)	0.210		
CA-AKI (%)	2.482 (1.154,5.341)	0.020	2.114(0.937,4.770)	0.071	22.207(4.066,121.271)	< 0.001	9.170(1.168,71.990)	0.035	16.607(2.774,99.423)	0.002	8.228(1.008,67.146)	0.049
NT-proBNP (per 100 pg/ml)	1.009 (1.005,1.013)	< 0.001	1.008(1.003,1.012)	0.001	1.018(1.011,1.024)	< 0.001	1.013(1.004,1.022)	0.006	1.015(1.007,1.023)	< 0.001	1.011(1.000,1.022)	0.044

*MACEs* major adverse cardiac events, *NT-proBNP* N-terminal pro-B-type natriuretic peptide, *CA-AKI* contrast-associated acute kidney injury

## Discussion

In this study cohort with STEMI patients undergoing pPCI, we found that the patients with lower creatinine were more likely to occur CA-AKI. The incidence of CA-AKI and creatinine levels at different time points were not significantly different between the traditional hydration group and the modified hydration group. CA-AKI have a potential predictive value for all-cause death and cardiac death at the follow-up period. Mitigating the occurrence of CA-AKI in STEMI patients may reduce post-pPCI all-cause death and cardiac death, and improve clinical outcomes.

The incidence of CA-AKI varies greatly after pPCI depending on the baseline demographic, clinical characteristics, diagnostic criteria and angiographic circumstances [5, 16]. Our study showed the incidences of CA-AKI were 8.7%. The main mechanism of CA-AKI includes contrast agent renal tubular toxicity and renal hemodynamic changes resulting in medullary hypoxia stress [9, 17]. Cholesterol micro-embolism also can induce acute renal dysfunction, which was a rare and subtle complication of invasive endovascular procedures. Inflammation may be the mechanism [18]. Hydration can increase blood volume, inhibit the activation of the renin–angiotensin–aldosterone system (RAAS) and the feedback of renal tubules, and dilute the concentration of contrast to prevent renal injury [19]. For our study, it’s the first time to forward new hydration that is based on the biphase of renal hemodynamics and the pharmacokinetics of contrast agent. Compared with the traditional hydration, the modified hydration relatively reduced the incidence of CA-AKI in STEMI patients after pPCI, although there was no statistically different. Trails with large samples are further needed.

The high level of NT-proBNP was responsible for the occurrence of CA-AKI in our study. NT-proBNP is secreted in response to increased volume or pressure overload, or myocardial ischemia [20, 21]. Clinical observations showed patients with heart failure had a decreased renal blood flow as a result of neurohormonal activation and inflammation, increased oxidative stress further compound these pathophysiologic mechanisms [9, 22]. The activation of RAAS and the sympathetic nervous system can further stimulate NT-proBNP[23]. In addition, inflammation may play an important role in the initiation and extension phases of CA-AKI. Inflammatory cells infiltrate the interstitium of the kidney via cytokines produced by renal endothelial cells, and cause the release of oxygen radicals, vasoconstrictors, and thromboxanes, leading to kidney damage [24, 25]. STEMI patients had increased systemic inflammatory responses. Therefore, WBC count was a risk factor for developing CA-AKI. Interestingly, we found lower creatinine levels may more likely to occur CA-AKI in STEMI patients undergoing pPCI. A previous study showed that patients who had contrast material in the course of cardiac catheterization may undergo procedural complications that can affect renal perfusions, such as arrhythmias, MI, and other vascular complications that do not occur to the same degree after vein contrast injections [26]. Therefore, the STEMI patients were particularly likely to have concurrent comorbidities that affect renal function. CA-AKI may be a diagnosis of misattribution or represents a condition obscured by other factors contributing to acute kidney injury. Other conditions caused by the entire range of conditions, treatments, and laboratory variations may also alter creatinine levels. These may affect the association between creatinine and CA-AKI. These results question the utility of creatinine as a marker for CA-AKI. Previous studies observed a reduction in the rise of creatinine after angiography withholding ACEI/ARB [27, 28]. However, a reduction in eGFR and increase in creatinine is known to occur with ACEI/ARB. The contrast medium can increase natriuresis due to its osmotic effect. These results in an activation of the glomerular tubule feedback with vasoconstriction of the afferent arteriole. Concomitant RAAS inhibitor therapy can cause pathological reduction of eGFR [29]. Based on the inconsistent results, we further assessed the significance of RAAS inhibition to CA-AKI, the multivariable result was not statistically significant.

We found that CA-AKI had a predictive value for all-cause death and cardiac death, that consistent with previous studies [12, 30, 31]. The relationship between CA-AKI and mortality strengthened as creatinine times above baseline increased. Greater incidence of procedural cardiac complications, including MI, elevated creatine kinase, hypotension and cardiac arrest in CA-AKI patients may be the reason [32]. In addition, the total ischemic time didn’t show statistics difference between the two hydration groups and between the CA-AKI group and no CA-AKI group, which had been proved to be associated with mortality in patients with STEMI undergoing pPCI [11]. To sum up, although there was no statistical difference, the modified hydration relatively reduced the incidence of CA-AKI and would be a benefit to the prognosis in STEMI patients undergoing pPCI. Therefore, trials with large samples are needed in future research.

## Study limitations

Despite the precise randomization, which results in an unimportance difference in baseline characteristics of the two groups, our study has several limitations. First, although our study had a relatively large sample size, the single-center study was also a limitation. Second, although many potential interfering covariables were adjusted by our models, we cannot rule out residual confounding factors or potential selection bias. Third, creatinine as the CA-AKI diagnostic indicator is insensitive, the pre-PCI serum creatinine levels may not be the real baseline serum creatinine level because of possible unstable clinical situations. However, our definition of CA-AKI is dependent on serum creatinine, which is an incomplete marker for kidney function. Fourth, few patients with renal insufficiency were included (56/438), this will affect the modified hydration effect.

## Conclusion

According to the findings, although this difference was not statistically significant, the modified hydration also relatively reduced the incidence of CA-AKI after pPCI. CA-AKI may be related to lower creatinine, WBC count and NT-proBNP. The relationship between CA-AKI and mortality strengthened as creatinine times above baseline increased. Therefore, modified hydration may be effective in preventing CA-AKI and improving long-term clinical outcomes. Further studies with a larger sample size are recommended.

## Supplementary Information

Below is the link to the electronic supplementary material.

Supplementary file1 (DOCX 168 KB)

## Data Availability

The datasets used and/or analyzed during the current study are available from the corresponding author on reasonable request.

## Abbreviations

BMI: Body mass index
SBP: Systolic blood pressure
DBP: Diastolic blood pressure
MI: Myocardial infarction
CHD: Coronary heart disease
OMI: Old myocardial infarction
WBC: White blood cells
hsCRP: Hypersensitivity C-reactive protein
eGFR: Estimated glomerular filtration rate
HbA_1_C: Glycosylated hemoglobin
TC: Total cholesterol
TG: Triglyceride
LDL-C: Low density lipoprotein cholesterol
HDL-C: High density lipoprotein cholesterol
pCKMB: Peak value of creatine kinase isoenzyme-MB
pTNI: Peak value of troponin I
pNT-proBNP: Peak value of N-terminal pro-B-type natriuretic peptide
LVEF: Left ventricular ejection fraction
LM: Left main trunk
pPCI: Primary percutaneous coronary intervention
STEMI: ST-segment elevation myocardial infarction
PCI: Percutaneous coronary intervention
MACEs: Major adverse cardiovascular events
CA-AKI: Contrast-associated acute kidney injury
