# Supplementary material for: Role of modified hydration for preventing contrast-associated acute kidney injury in patients with ST-segment elevation myocardial infarction after primary percutaneous coronary intervention
Source: Intern Emerg Med. 2022 Dec 20;18(1):67–76. doi: 10.1007/s11739-022-03109-3 (PMC9883347; doi:10.1007/s11739-022-03109-3)
Supplement: Supplementary file 1 — Supplementary file1 (DOCX 168 KB) [file 11739_2022_3109_MOESM1_ESM.docx]

**Supplementary Figure 1. Flow chart.**

**
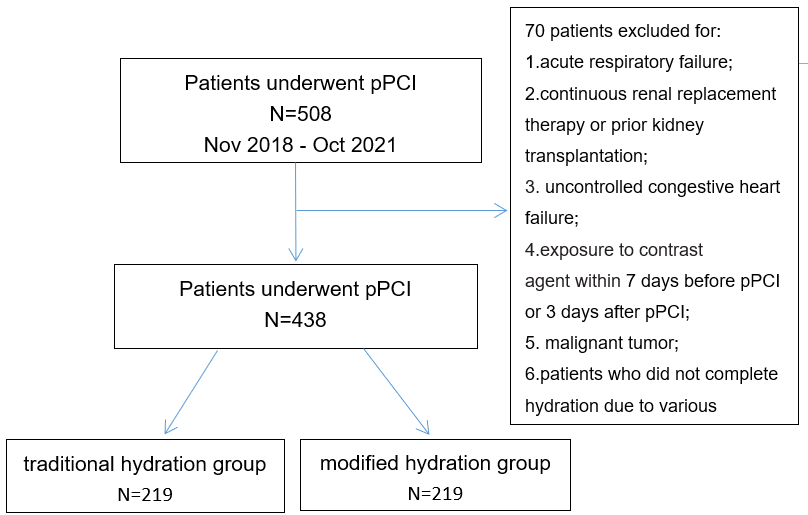
**

pPCI, primary percutaneous coronary intervention.

**Supplementary Figure 2. The changes of creatinine (A) and eGFR (B) in different hydration groups.**

**
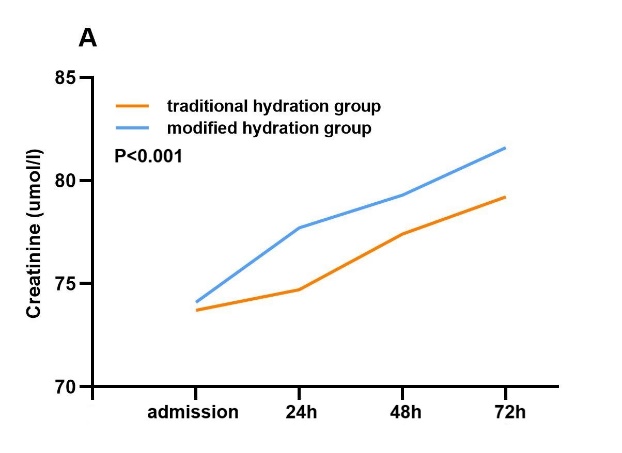

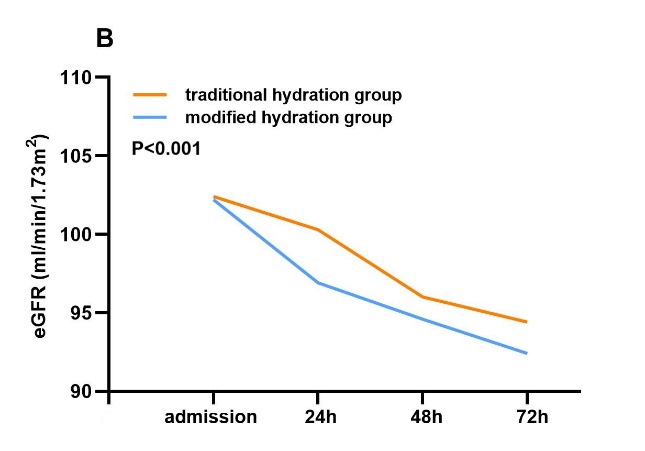
** eGFR, estimated glomerular filtration rate.

**Supplementary Figure 3. The cumulative incidence of different creatinine grades.**

**
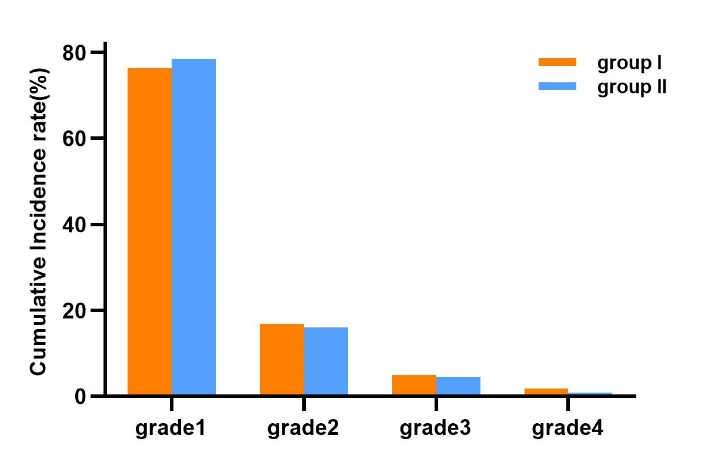
**

Group I represent traditional hydration; group II represent modified hydration.

Grade1 was characterized by an increase in serum creatinine of <1.25 times increase above baseline; grade 2 was 1.25 to 1.4 times increase above baseline; grand 3 was 1.5 to 1.9 times increase above baseline; grade 4 was >2.0 times increase above baseline.

**Supplementary Table 1. Clinical characteristics in CA-AKI and no CA-AKI group.**

|  | CA-AKI | No CA-AKI | P value |
| --- | --- | --- | --- |
|  | *N=*38 | *N=*400 |  |
| Male (%) | 21(55.3) | 316(79.0) | 0.001 |
| Age(years) | 63.4±16.2 | 60.6±12.2 | 0.305 |
| BMI (kg/m^2^) | 25.9±3.9 | 25.7±3.5 | 0.690 |
| SBP (mmHg) | 132.5±20.5 | 127.0±23.0 | 0.155 |
| DBP (mmHg) | 77.6±15.0 | 77.4±15.7 | 0.966 |
| Heart rate(bpm) | 78(65,88) | 75(63,86) | 0.531 |
| Anterior MI (%) | 17(44.7) | 186(46.5) | 0.835 |
| Killip II-IV (%) | 4(10.5) | 39(9.8) | 0.779 |
| Cardiogenic shock (%) | 3(7.9) | 23(5.8) | 0.484 |
| Medical history |  |  |  |
| Hypertension (%) | 25(65.8) | 240 (60.0) | 0.485 |
| DM (%) | 15(39.5) | 109(27.3) | 0.110 |
| CHD (%) | 1(2.6) | 23(5.8) | 0.710 |
| OMI (%) | 0(0) | 10(2.5) | 0.447 |
| Dyslipidemia (%) | 16(42.1) | 183(45.8) | 0.666 |
| Current/ex-smoker（%) | 17(44.7) | 271(67.8) | 0.004 |
| Pharmacotherapy before admission | |  |  |
| Antiplatelet agent, % | 7(18.4) | 40(10.0) | 0.163 |
| ACEI/ARB, % | 19(50.0) | 199(49.8) | 0.977 |
| β-blocker, % | 2(5.3) | 33(8.3) | 0.756 |
| Statins, % | 16(42.1) | 183(45.8) | 0.666 |
| Metformin, % | 3(7.9) | 30(7.5) | 1.000 |
| Laboratory values |  |  |  |
| WBC(×10^9^/L) | 11.6(9.4,13.1) | 9.1(7.4,11.4) | <0.001 |
| Hemoglobin (g/l) | 135.3±22.6 | 139.9±18.8 | 0.239 |
| HsCRP(mg/l) | 11.5(4.2,26.8) | 5.3(2.2,15.0) | 0.013 |
| Creatinine (umol/l) | 66.0±24.9 | 74.7±27.1 | 0.059 |
| eGFR (ml/min/m^2^) | 111.2±59.2 | 101.5±37.5 | 0.325 |
| HbA_1_C (%) | 6.6(5.6,9.0) | 6.0(5.5,7.0) | 0.036 |
| TC (mmol/l) | 4.3(3.8,5.1) | 4.6(3.9,5.4) | 0.350 |
| TG (mmol/l) | 1.4(1.1,1.9) | 1.5(1.1,2.2) | 0.463 |
| LDL-C(mmol/l) | 2.6(2.0,3.1) | 2.8(2.3,3.2) | 0.541 |
| HDL-C (mmol/l) | 1.0(0.8,1.2) | 1.0(0.9,1.1) | 0.938 |
| pCKMB(ng/ml) | 134.5(47.8,239.3) | 97.1(38.5,209.0) | 0.298 |
| pTNI(ng/ml) | 50.0(26.6,50.0) | 44.1(15.4,50.0） | 0.049 |
| pNT-proBNP(pg/ml) | 2170.0 (857.0,4240.5) | 1342.0(645.0,2674.0) | 0.038 |
| Angiography values |  |  |  |
| LM (%) | 1(2.6) | 26(6.5) | 0.496 |
| Triple-vessel (%) | 239(60.5) | 226(56.5) | 0.632 |
| PCI (%) | 37(97.4) | 381(95.3) | 0.577 |
| Volume (ml) | 110(110,150) | 110(110,130) | 0.432 |
| LVEF | 0.56(0.48,0.62) | 0.58(0.51,0.63) | 0.197 |
| Total ischemic time (hour) | 4.5(2.0,7.0) | 4.5(3.0,6.0) | 0.944 |
| TIMI score | 4.5±2.3 | 4.0±2.0 | 0.156 |
| GRACE score | 150.3±42.1 | 145.5±28.1 | 0.489 |
| SYNTAX score | 17.0±5.0 | 16.0±6.4 | 0.338 |
| α_1_-microglobulin (mg/dl) | 1.3(0.6,3.2) | 1.0(0.6,1.9) | 0.046 |
| Microalbumin (mg/dl) | 1.9(1.1,10.9) | 1.3(1.1,3.8) | 0.195 |
| Intake of fluids in 24h(ml） | 2142.9±652.5 | 2317.9±580.7 | 0.185 |
| Urine volume in 24h (ml) | 1675.5±661.0 | 1870.6±722.0 | 0.225 |

BMI, body Mass Index; SBP, systolic blood pressure; DBP, diastolic blood pressure; MI, myocardial infraction; DM, diabetes mellitus; CHD, coronary heart disease; OMI: old myocardial infarction; WBC, white blood cells; hsCRP, hypersensitivity C-reactive protein; HbA1C, glycosylated hemoglobin; TC, total cholesterol; LDL-C, low density lipoprotein cholesterol; HDL-C, high density lipoprotein cholesterol; eGFR, estimated glomerular filtration rate; ACEI, angiotensin-converting enzyme inhibitor; ARB, angiotensin II receptor blocker; NT-proBNP, N-terminal pro-B-type natriuretic peptide; CKMB, creatine Kinase Isoenzyme-MB;TNI, troponin I;LM, left main trunk; PCI, percutaneous coronary intervention; LVEF, left ventricular ejection fraction; CA-AKI, contrast-associated acute kidney injury.

Total ischemic time represent the time from symptom initiation to reperfusion.

**Supplementary Table 2. Clinical events during the follow-up period.**

|  | Group I | Group II | P value |
| --- | --- | --- | --- |
| MACEs (%) | 25(11.4) | 20(9.1) | 0.431 |
| All-cause death (%) | 5(2.3) | 1(0.5) | 0.216 |
| Cardiac death (%) | 4(1.8) | 1(0.5) | 0.372 |
| Non-fatal MI (%) | 8(3.7) | 7(3.2) | 0.793 |
| Revascularization (%) | 13(5.9) | 6(2.7) | 0.101 |
| Cardiac rehospitalization (%) | 16(7.3) | 12(5.5) | 0.435 |
|  | CA-AKI | No CA-AKI | P value |
| MACEs (%) | 8(21.1) | 37(9.3) | 0.043 |
| All-cause death (%) | 4(10.5) | 2(0.5) | 0.001 |
| Cardiac death (%) | 3(7.9) | 2(0.5) | 0.005 |
| Non-fatal MI (%) | 2(5.3) | 13(3.3) | 0.380 |
| Revascularization (%) | 2(5.3) | 17(4.3) | 0.676 |
| Cardiac rehospitalization (%) | 3(7.9) | 25(6.3) | 0.725 |

MACEs, major adverse cardiac events; MI, myocardial infraction. CA-AKI, contrast-associated acute kidney injury.

Group I represent traditional hydration; group II represent modified hydration.
